# Shockwaves from air bubbles within pits induced by nearby cavitation bubbles

**DOI:** 10.1016/j.ultsonch.2025.107602

**Published:** 2025-10-03

**Authors:** Jie Li, Siyu Chen, Jing Luo, Weilin Xu, Jiguo Tang, Tong Qu

**Affiliations:** a State Key Laboratory of Hydraulics and Mountain River Engineering, Sichuan University, Chengdu 610065 China; b Hydraulics Department, Changjiang River Scientific Research Institute, Wuhan 430010 China

**Keywords:** Cavitation bubble, Air bubble, Pit, Microjet, Shockwave

## Abstract

The cavitation intensity in near-boundary regions was significantly affected by the smoothness of boundaries. Micro pits on these boundaries may harbor smaller air bubbles or gas nuclei, and cavitation bubbles within cavitation clouds or cavitation bubble clusters inevitably interact with air bubbles in these pits. In this study, the experimental setup employed underwater Corona Discharge to generate controlled cavitation bubbles, and experimental observations were made with high-speed photography system. The experimental results revealed that for a given pit spatial size (*ξ*), the presence of air bubbles within pits reduces the evolution period of cavitation bubble (defined as the ratio of the time from cavitation bubble inception to its first collapse to the Rayleigh time) as the dimensionless bubble-boundary distance (*γ*) increases. Additionally, compared to scenarios without air bubbles, the evolution period of cavitation bubbles decreases, while the velocity of microjet increases. The cavitation bubble shockwave pressure follows a distinct pattern as *γ* increases: it initially decreases, followed by an increase, and eventually stabilizing. Within the *γ* range of 0.9 to 1.7, the air bubbles in pits significantly attenuate the shockwave pressure generated during cavitation bubble collapse (air bubble can reduce cavitation bubble collapse pressure by up to 80 %). Through the assistance of Schlieren techniques, a novel ‘cavitation’ behavior of air bubble within the pits was discovered. The phenomenon is characterized by the generation of an ‘implosion shockwave’ during the air bubble collapse (the propagation speed of this ‘shockwave’, as observable in high-speed images, is approximately 1534 ± 39 m/s, which is on the order of the speed of sound in the liquid, around 1500 m/s). Further analysis revealed the critical conditions for the ‘implosion shockwave’ from the small air bubbles within pits induced by nearby cavitation bubbles. Specifically, the critical dimensionless standoff distance(γ*) exhibits an exponential decay with increasing pit spatial size (*ξ*), and the coefficient is likely related to the ratio of maximum bubble radii (*R*_air_/*R*_max_) between the air bubble and cavitation bubble. These innovative findings offer valuable references for controlling and evaluating cavitation intensity in defective water flow boundaries.

## Nomenclature

**Notation**
Symbols: Symbol Meaning. Unit
*A*: Cavitation bubble projected area. mm^2^
*d*: Bubble-wall distance. mm
*H*: Pit depth. mm
*L*_s_: Distance of bubble centroid to *P*_1_. mm
*p*: Local environmental pressure. kPa
*P*_1_: Pressure measurement point
*P*_v_: Saturation pressure within the cavitation bubble. kPa
*r*_air_: Initial radius of air bubble. mm
*R*: Equivalent radius of cavitation bubble. mm
*R*_air_: Maximum radius of the air bubble. mm
*R*_max_: Maximum radius of the cavitation bubble. mm
*t*: Evolution time of cavitation bubble. μs
$T_{o}$: Rayleigh time. μs
t*: Dimensionless evolution time of cavitation bubble
*V*: Velocity of microjet. m / s
*W*: Pit width. mm
Δp: Pressure difference of the bubble. kPa
Δt: Temporal duration spanning from cavitation bubble nucleation to initial collapse. μs
*γ*: Dimensionless distance of bubble-wall
Γ: Dimensionless distance of bubble-sensor
*ξ*: Pit spatial size
ρ: Density of water. kg / m^3^
τ*: Dimensionless evolution period
*φ*: Dimensionless pit diameter
*η*: Dimensionless pit depth

## 1 Introduction

The smoothness of boundaries significantly influences near-boundary cavitation intensity. Surface imperfections, such as construction defects or cavitation erosion pits in concrete [1], can trap air bubbles or gas nuclei [2,3]. Although the interaction between air bubbles and cavitation bubbles has been reported to mitigate wall erosion [4,5], cavitation itself originates from the expansion and collapse of gas nuclei under low pressure [6], influenced by gas content [7]. This raises a question that whether pit trapped small air bubbles mitigate erosion or exhibit cavitation-like behavior? Therefore, studying the interaction between cavitation bubbles and small air bubbles within pits is essential for accurately evaluating erosion risks from surface irregularities and optimizing air bubble sizes for resistance cavitation erosion techniques.

The effect of pits on cavitation bubble dynamics and cavitation erosion has been widely studied. Cylindrical holes can induce secondary cavitation and vortex rings during bubble collapse [8]. Požar 2021 [9] also observed that the collapse of a laser-induced cavitation bubble near a concave surface produces shockwaves that lead to secondary cavitation at the acoustic focus. Furthermore, the reflection and focusing of collapse shockwaves can trigger the violent collapse of surrounding cavitation bubbles [10]. In addition, the pits alter the local flow field and cause pressure fluctuations [11]. Trummler et al. [12] quantitatively show that the collapse pressure of a laser-induced bubble near crack geometries (*R*_c_/*R*_max_ = 0, 0.5, 0.75) can be up to 50 % higher than near a flat wall. Liu et al. [13] revealed that needle-like microjets impacting a pitted wall can reach velocities more than 10 times higher than conventional microjets, accompanied by complex energy transfer and dissipation mechanisms. Moreover, shockwaves is also a critical factor induce cavitation erosion [14,15]. When a bubble near a rigid boundary forms a re-entrant jet, it transitions into a vortex ring-like structure, generating significantly higher pressures [16], which is considered a major mechanism contributing to wall damage [17,18]. However, some studies suggest that a certain degree of surface roughness may reduce cavitation erosion, larger pit curvature can decrease microjet velocity and reduce overall momentum [[19], [20], [21]]. Kadivar et al. [22] further suggested that surface roughness, such as V-shaped riblets, can reduce the formation of torus rings during bubble collapse, thereby mitigating cavitation erosion risk. While existing studies have predominantly focused on scenarios where pit dimensions larger than the cavitation bubble radius, research on the collapse dynamics near different size pits remains limited.

In addition to studying the effects of boundary geometry, numerous researchers have focused on exploring effective strategies to resist cavitation erosion. A common approaches involving controlling the direction and intensity of cavitation bubble collapse. For instance, applying elastic materials can promote the transformation of cavitation bubbles into a ‘mushroom’ shape, which subsequently splits into two parts moving toward and away from the boundary, thereby reduced the peak collapse pressure [23,24]. However, the necking phenomenon during the collapse of such ‘mushroom-cap’ bubbles can generate localized high-pressure zones and accelerate microjet development, which may counteract erosion resistance [13,25]. Furthermore, under the influence of a free surface, cavitation bubbles tend to move away from the free surface [26,27]. Consequently, studies have also examined how the quantity, concentration, and size of air bubbles influence cavitation erosion on boundaries [4,5]. From a mesoscopic perspective, the mechanisms by which air bubbles mitigate cavitation damage involve two main aspects. Firstly, air bubbles can alter the direction of cavitation bubble collapse [[28], [29], [30]], with the microjet direction being influenced by the ratio of oscillation times between the cavitation bubble and the attached air bubble [31]. Secondly, energy transfer between the air bubble and the cavitation bubble can modify the energy conversion during collapse [32]. A more intrinsic mechanism involves air bubbles inducing shockwave stratification during cavitation bubble collapse, thereby reducing its intensity [33]. The mitigating effect depends strongly on the number, size, and distance of air bubbles relative to the cavitation bubble [34,35]. Although air bubbles generally alter collapse direction and intensity, studies of laser-induced cavitation near horizontal plates (*r*_air_/*R*_max_≈0.8–1.1) have revealed that air bubbles can exhibit four distinct morphologies (Omega, hemisphere, hemisphere to hat with split, and hemisphere to hat without split), along with three jetting modes: initial, multiple, and delayed jets [36]. Guo et al.,2025[37] observed that during interactions between air and cavitation bubbles (*r*_air_/*R*_max_≈0.2–1.8), the air bubble also generates a microjet directed toward the wall (Jet_air_). Wang et al. [38] reported that oscillations of an air bubble (*r*_air_≈3 mm) attached to an elastic membrane during cavitation bubble collapse (*R*_max_≈5 mm), which increase system energy and membrane deformation, potential aggravation of boundary erosion. In a word, existing studies have primarily focused on interactions in free fields or near smooth walls. Rough surfaces can not only cause asymmetric collapse of cavitation bubble but also trap air bubble, significantly altering cavitation bubble dynamics [39]. Although biomimetic structures [40] and gas-entrapment holes [[41], [42], [43]] can redirect jets and promote cavitation bubble collapse away from walls, the behavior of air bubbles trapped in pits remains unclear. A key unresolved question is whether these trapped air bubbles simply attenuate collapse pressure or could themselves evolve into damaging shockwave sources under specific conditions.

Based on the above literatures review, it can be seen that research on the interactions between small air bubbles or gas nuclei trapped within pits on water flow boundaries and cavitation bubbles in cavitation clouds or clusters is currently exceedingly scarce. To address this gap, this study employed underwater Corona Discharge to generate cavitation bubble, coupled with a high frequency transient pressure testing system and high-speed photography system, to systematically explore the interplay of small air bubbles in pits with cavitation bubbles. The study focuses on two main parts: the role of small air bubbles in the pits in affecting cavitation bubbles, and the effect of cavitation bubbles on small air bubbles in the pits. An innovative ‘cavitation’ behavior of small air bubbles in those pits under the effect of cavitation bubbles was discovered, and the critical conditions for the occurrence of the ‘implosion shockwave’ phenomenon of small air bubble in the pit were summarized.

## 2 Experimental methods

**Fig. 1:**
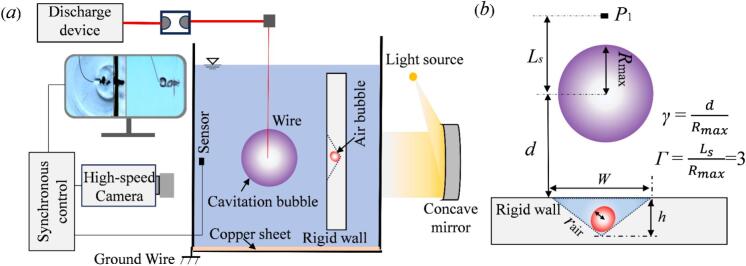
Schematic diagram of experimental setup.

The cavitation bubbles were generated by an underwater Corona Discharge system, combined with high frequency transient pressure testing system and high-speed camera system to observe the interplay of small air bubbles in pits with cavitation bubbles. The experimental setup is shown in Fig. 1.

The underwater Corona Discharge equipment converts a conventional 220 V AC power supply into a discharge voltage of 0–33 kV by a voltage transformer. The high-voltage was stored in a capacitor before being applied to the discharge electrode. The discharge electrode in the experiment is a 0.3 mm diameter single-core stainless steel wire, insulated with a coating, which submerged in a liquid medium with a conductivity of 12.65mS/cm (temperature 23 ± 2 °C). A copper grounding electrode was placed at the tank bottom. After triggering the discharge switch, the liquid at the electrode tip was broken down to form a plasma, which evolved into a cavitation bubble [44], which is highly repeatable [45].

A high-speed camera (Photron Inc. Fastcam SA-Z, maximum frame rate of 1,000,000 fps) with a prime lens (Nikon Inc.; AF-S 85 mm f/1.4G) was used to record the cavitation bubble evolution process. The resolution is 256 × 256 pixels (spatial resolution 0.2273 mm/pixel), and the frame rate is 180,000 fps. Two optical configurations were utilized: (1) Shockwave optical path: exposure time of 0.25 μs, using Schlieren technique to illuminate the path [46], to capture the shockwave morphology during cavitation bubble collapse; (2) Microjet optical path: exposure time extended to 3.95 μs, using an LED flat-panel light source to illuminate from the opposite side of the high-speed photography system to capture microjet development process during cavitation bubble collapse.

A high-frequency piezoresistive pressure sensor (Test Electronics Information Co. Ltd, CY400) with a 20 MPa measurement range and ±0.25 % full-scale accuracy was employed to capture cavitation bubble collapse shockwave pressures. The sensor chip radius is 1.5 mm, and 20 MHz sampling frequency, the data length is 4 M, a maximum response frequency of 500 kHz and a rise time of 0.2 μs, synchronized with the discharge signal trigger. The suitability of this pressure sensor for measuring the pressure of cavitation bubble collapse shockwaves has also been validated in previous studies [47].

The pit in this experiment was pre-fabricated on the surface of transparent acrylic plate. The pit features an inverted triangular conical geometry defined by its width (*W*) and depth (*H*). The position of pressure measurement and the parameters used in the study are defined in Fig. 1(b). The radius of cavitation bubble is determined using the equivalent radius $R=\sqrt{A/\pi }$, *A* is the cavitation bubble projected area from high-speed images, which has the equivalent maximum radius, $R_{\max}$, was 10.8 ± 0.5  mm. *P*_1_ denotes the pressure measurement location. The dimensionless bubble-sensor distance is defined as $\Gamma =L_{s}/R_{\max}=3$, where *L*_s_ is the displacement from the bubble centroid to *P*_1_. The dimensionless bubble-wall distance is $\gamma =d/R_{\max}$, where *d* is the distance from the bubble centroid to the boundary. The dimensionless pit diameter and dimensionless pit depth are defined as *φ* = $W/R_{\max}$and *η* $=H/R_{\max}$. An initial radius of air bubble (*r*_air_) about 1.5–2.5 mm (*r*_air_/R_max_ = 0.2–0.25), the air bubble was injected into the pit using a 1 mL syringe. It is important to note that although the air bubbles within the pit are influenced by buoyancy and appear elongated, this study does not involve changing the size of the air bubble. After controlling the air bubble volume to be consistent, we used the equivalent radius of a sphere with the same volume to calculate *r*_air_. This approach reasonably reflects the size of the air bubble. Furthermore, numerous preliminary experiments show that under the influence of a nearby cavitation bubble, the air bubble always evolve and move along the centerline connecting the air bubble and cavitation bubble. This indicates that the interaction between the cavitation bubble and the air bubble is far greater than the effect of buoyancy on the air bubbles. Therefore, buoyancy’s effect on the air bubbles were neglected in this study. The experimental conditions are shown in Table 1. To ensure the accuracy and repeatability, each experimental condition was repeated 5–8 times.

**Table 1:** Experimental conditions.

*W* (mm)	*h* (mm)	*r*_air_ (mm)	*γ* = *d/R*_max_	*φ = W/R*_max_	*η = H/R*_max_	*ξ = W/H*
2	2; 4; 6; 8; 11	0; 1.5–2.5	0.6–3	0.185–2.95	0.185–1.01	0.18–8
4						
6						
8						
11						
15						
22	4;6;8;11	0; 1.5–2.5	0.6–3	0.185–2.95	0.185–1.01	0.18–8
32						

## 3 Effect of small air bubble in pit on cavitation bubble collapse

Existing studies focuses on the mechanism by which air bubbles weaken the collapse energy of cavitation bubble in free-field or near flat walls [33], thereby reduced cavitation erosion damage [4,5]. However, the effects of small air bubbles trapped in pits are poorly understood. Therefore, the effects of small air bubbles (*r*_air_ = 1.5–2.5 mm) in pits on the cavitation bubble evolution periods, microjet, and implosion shockwave characteristics are analyzed in this section.

### 3.1 Influence of small air bubble within pit on period of cavitation bubble

**Fig. 2:**
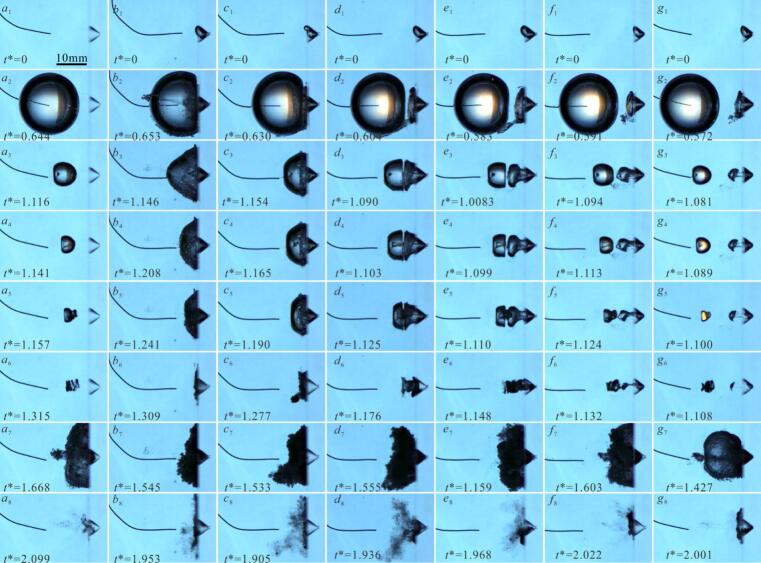
Effect of air bubble within pit on evolution of cavitation bubble.

Fig. 2 shows the influence of small air bubbles within a pit (with a depth of η = 0.37 and a diameter of φ = 0.55) on the cavitation bubbles evolution under different dimensionless distance γ. In Fig. 2(a), γ = 1.2, without an air bubble in the pit. At t* = 0.644 (where t* is calculated using Equation 1 [48]), *R*_max_ = 10.79, and exhibiting roughly spherical shape (Fig. 2(a_2_)). During collapse, the cavitation bubble’s far-side surface contracts at 72.41 m/s compared to 15.52 m/s on the pit-proximal side, resulting in the formation of a microjet directed toward the pit. The peak microjet velocities reach 57.13 m/s (as shown in Fig. 2(a_3_-a_4_)), while bubble volume minimization occurs at t* = 1.315 without directly impacting the pit (Fig. 2(a_6_)). Subsequently, the cavitation bubble rebounds and spreads along the pit and the adjacent wall, forming a ‘pancake’ shape (Fig. 2(a_7_)). During the subsequent contraction to minimum radius, the rebound cavitation bubble contracts inwards the interior of the pit.

(1) $$t*=t/T_{o}$$

(2) $$T_{o}=1.83R_{\max}\sqrt{\rho /\left(p-P_{v}\right)}$$

here, the variable *t* represents physical time, calculated from frame interval data of the high-speed camera system, $T_{o}$ is the Rayleigh time, *p* is the local environmental pressure, approximately 95.5 kPa; and, ρ is the density of water. *P*_v_ is the saturation pressure inside of cavitation bubble. Different studies have used different values for *P*_v_. For instance, Gong et al. (2013) [49] used *P*_v_ = 0 kPa, whereas Zhang et al. (2016) [27] chose *P*_v_ = 20 kPa. In our data processing, calculations were performed using both *P*_v_ = 0 and *P*_v_ = 20 kPa. The results show that the difference in *P*_v_ primarily affects the value but does not alter the trends in the data (see Supplementary Material, Fig. S2). Given the difficulty in accurately measuring *P*_v_, we simplified our approach by using *P*_v_ = 0 kPa for subsequent analysis.

In Fig. 2(b), *R*_max_ = 10.29 mm, *γ* = 0.72, with an air bubble (*r*_air_≈1.5 mm) was trapped into the pit (Fig. 2(*b*_1_)). During the expansion of the cavitation bubble, its volume increases, driving the surrounding fluid to move, thereby compressing the air bubble within the pit. However, the volume of air bubble is larger than the volume of pit, when it is squeezed by cavitation bubble, the pit becomes insufficient to accommodate air bubble, causing it to rupture and release gas. The subsequent liquid motion during cavitation bubble evolution entrains gas into the cavitation bubble interior (gas entrainment) (Fig.2b2). As the cavitation bubble contracts, the air bubble is drawn further inward, simultaneously increasing the non-condensable gas concentration inside the cavitation bubble, result in the microjet less visible (Fig. 2(*b*_3_-*b*_5_)), which matches the findings of previous studies [33]. As the cavitation bubble contracts to its minimum volume, it spreads out over the surface of pit (Fig. 2(*b*_6_)). In Fig. 2(c), *r*_air_≈1.5 mm, *R*_max_ = 10.60 mm, *γ* = 0.81, the air bubble was also subjected to compression during the expansion of cavitation bubble. However, there is a gap between the cavitation bubble and the pit surface, the air bubbles can spill out along the pit surface after expanding and rebounding. Consequently, the air bubble does not rupture or being entrained into the cavitation bubble interior (Fig. 2(*c*_2_)). The contracting cavitation bubble interface near the pit gradually incorporates the air bubble. Since the amount of entrapped gas was relatively small, microjet formation and development were not significantly affected (Fig. 2(*c*_3_-*c*_4_)). After penetrating the opposite surface of the cavitation bubble, the microjet continues its propagation and fully interacted with the air bubble. The air bubble subsequently acts as an energy-absorbing buffer, effectively preventing microjet direct impingement on the pit interior (Fig. 2(*c*_5_)). Following the microjet continue to development and impact on the inner wall of pit, a rebound phenomenon occurs (Fig. 2(*c*_6_)). The direction of rebounding cavitation bubble is opposite to the microjet impact direction (Fig. 2(*c*_7_)). Fig. 2(d) shows the cavitation bubble evolution near *η* = 0.37, *φ* = 0.55, *γ* = 0.99, with *R*_max_ = 11.1 mm, and its interaction with an air bubble (*r*_air_≈1.5 mm). The cavitation bubble evolution progress closely resembles that shown in Fig. 2(c). During cavitation bubble contraction, the air bubble stretches longitudinally (axial length: ∼7.76 mm) along the pit’s central axis. When the cavitation bubble collapse, the elongated air bubble is sufficient to buffer microjet. During the cavitation bubble rebound, it converges towards the interior of pit, and the shape of rebounding cavitation bubble is exhibits ‘winged’ (Fig. 2(*d*_8_)).

As *γ* increases further to 1.15, a distinct gap remains between the cavitation bubble at its maximum volume and the air bubble, indicating incomplete contact between them (Fig. 2(e)). As the cavitation bubble contraction, the air bubble was elongated and exhibited a necking phenomenon. Concurrently, a microjet directed towards the pit was observed (Fig. 2(*e*_3_-*e*_4_)). During this phase, the elongated air bubble’s buffering and merging effect on the microjet becomes more pronounced (Fig. 2(*e*_5_)). The cavitation bubble reached its minimum volume at t* = 1.148 (*e*_6_)). In Fig. 2(f), *r*_air_≈1.5 mm, *R*_max_ = 10.95 mm, *γ* = 1.35, The dynamics of cavitation bubble growth exhibit comparable characteristics to those in Fig. 2(e)*.* However, the cavitation bubble contraction and attraction the air bubble to progressively deform into a ‘dumbbell’ shape (Fig. 2(*f*_4_-*f*_6_)). Eventually, the air bubble split into two distinct parts: an exterior segment and a pit-bottom remnant. This bubble splitting occurs because the rapid contraction of the cavitation bubble generates strong local fluid flows. The air bubble was dragged by fluid motion, and towards the outside of the pit, while the air bubble inside pit remains in contact and adhered to the pit wall. When the attractive from the cavitation bubble is insufficient to overcome the air bubble’s adhesion to the pit wall, the air bubble undergoes a splitting phenomenon. Fig. 2(g) shows *r*_air_≈1.5 mm, *R*_max_ = 10.94 mm, *γ* = 1.62. At this larger *γ*, the increased distance from the cavitation bubble to the pit weakens the attraction acting on the air bubble entrapped by pit. Consequently, the influence of the small air bubble on the evolution process of cavitation bubble diminishes. Nevertheless, the air bubble still undergoes a splitting phenomenon into parts inside and outside the pit (Fig. 2(*g*_3_-*g*_6_)).

Fig. 2 shown significant variations in cavitation bubble-air bubble interaction dynamics within the pit across different *γ* regimes. Furthermore, pit-contained small air bubbles modulate the cavitation bubble’s evolution time, with t* declining from 1.309 to 1.108 across 0.72 ≤ *γ* ≤ 1.62 (Fig. 2(*b-g*)). A comparison with the case without an air bubble (Fig. 2(a)) under the same γ conditions, shows that entrapped air bubbles within the pit accelerate cavitation bubble collapse, reducing the contraction time to minimum volume by 12.7 %. This indicates that both *γ* and the air bubbles within pit influence the cavitation bubble evolution time. Therefore, we further analyzed how pits with air bubbles alter evolution time as a function of *γ*. The dimensionless evolution period ($\tau^{*}$) was calculated using in Equation (3):

(3) $$\tau^{*}=\Delta t/T_{o}$$

Here, Δt denotes the temporal duration spanning from cavitation bubble nucleation to initial collapse (first collapse time).

**Fig. 3:**
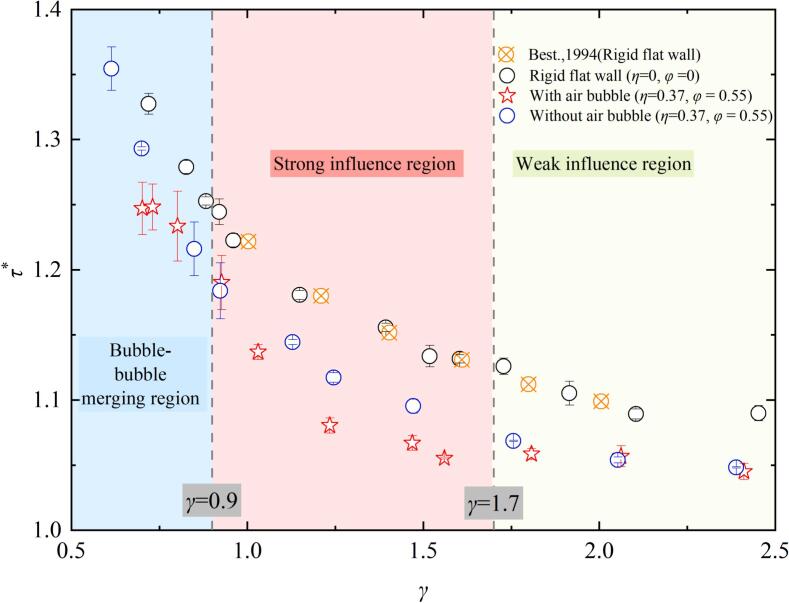
The relationship between the evolution period of cavitation bubble and the cavitation bubble- wall distance.

For a pit with depth *η* = 0.37 and diameter *φ* = 0.55, the evolution period of cavitation bubble near the pit is demonstrated by the blue  symbols in Fig. 3. τ* > 1 indicates that the first evolution period of the cavitation bubble exhibits temporal extension. It can be observed that τ* gradually decreases as *γ* increases. The evolution period (τ*) of cavitation bubble near the rigid flat wall (*η* = 0, *φ* = 0, as indicated by the black ‘⚪’in Fig. 3) is consistent with the experimental results reported by Best, 1994 [50]. However, compared to the rigid flat wall, τ* near the pit is slightly smaller, with the maximum reduction being about 5.9 %. The presence of the pit contributes to the reduction periods of bubble for two reasons: on the one hand, the water within the pit has better fluidity, allowing the surrounding fluid to rapidly refill the region released by the contraction of the cavitation bubble surface near the pit. On the other hand, the presence of the pit slightly increases the space of the bubble – boundary, thereby decreasing the constraining influence of the wall on the cavitation bubble. When small air bubbles (*r*_air_ = 1.5–2.5 mm) are present within pits. For *γ* < 0.9, the small air bubbles within the pit does not significantly shorten the cavitation bubble evolution period. The cavitation bubble temporal delay arises from merge with air bubbles, which introduces additional non-condensable gas content that retards the dynamic of cavitation bubble. However, for 1.7 > *γ* > 0.9, the cavitation bubble evolution period further decreases. Compared to the pit without air bubbles, τ* is reduced by about 3.6 %, and compared to the rigid flat wall, the reduction is approximately 7.8 %. The additional reduction in τ* occurs as entrapped air bubbles function as an infinitely small free surface during cavitation bubble contraction, and it is well known that the presence of a free surface shortens the cavitation bubble evolution period [51,52]. When *γ* exceeds 1.7, the influence of the air bubble on cavitation bubble dynamics gradually diminishes, resulting in nearly identical evolution periods for cavitation bubbles near pits without air bubble.

### 3.2 Influence of small air bubble within pit on microjet of cavitation bubble

In Section 3.1, our analysis focused on the characteristic timescales of cavitation bubble. Since τ* is related to the surface contraction velocity of cavitation bubble, we quantitatively characterized microjet dynamics by measuring the peak displacement velocity (*v*) during cavitation bubble collapse, and the dimensionless microjet velocity (v*) is obtained by $v/{\left(\Delta p/\rho \right)}^{0.5}$ [27,53], where $\Delta p=p-P_{v}$ and *ρ* is the water density.

**Fig. 4:**
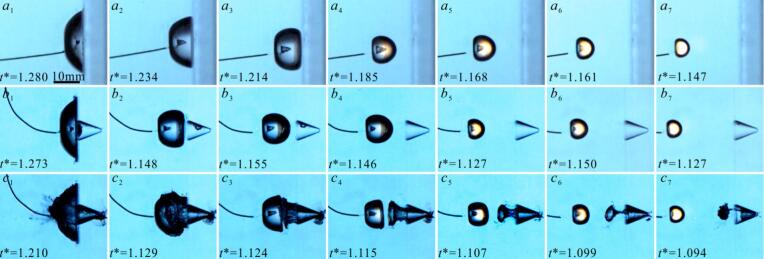
Effect of air bubble within pit on microjet of cavitation bubble.

Fig. 4(*a–c*) present microjet development under varying *γ* conditions for three configurations, such as rigid flat wall boundary, air-free pit (*η* = 0.74, *φ* = 0.55), and pit containing an air bubble (*η* = 0.74, *φ* = 0.55, *r*_air_≈2 mm), respectively. Under the condition of *γ* = 0.73, during collapse of cavitation bubble near the rigid boundary, a wall-directed conical microjet that propagates internally for 55 μs, reaching a velocity nearly 74 m/s (Fig. 4(*a*_1_)). The puncturing of the microjet leads to the cavitation bubble exhibiting a ‘ring-like’ shape. Fig. 4(*b*_1_) shows the collapse microjet morphology near the pit (*η* = 0.74, *φ* = 0.55), and the microjet morphology is similar to that in Fig. 4(*a*_1_). However, when a small air bubble was present within the pit, the cavitation bubble instantaneously merges with adjacent air bubble, increasing the non-condensable gas concentration. The presence of air bubble obscures microjet development during cavitation bubble collapse, while simultaneously inducing a conical of the cavitation bubble (as is shown the Fig. 4(*c*_1_)). At *γ* = 0.9, the microjet development time within the bubble increased to approximately 100 μs, and the microjet velocity rises to about 107.6 m/s (Fig. 4(*a*_2_)). However, when air bubbles are present within the pit, closely resembles Fig. 4(*c*_1_), it’s difficult to observe the development of the microjet (Fig. 4(*c*_2_)). Fig. 4(*a*_3_) shows complete wall attachment of the cavitation bubble, while its interface adjacent to air-free pits exhibits pronounced curvature (Fig. 4(*b*_3_)). This is because the water inside the pit weakens the constraint effects, promoting a more uniform shrinkage of the cavitation bubble. However, near the side of the pit with air bubble, the surface of the cavitation bubble was flat (Fig. 4(*c*_3_)). This occurred because the air bubble was pulled out of the pit, and forms a deformable boundary. The air bubble surface of moves following the contraction of the cavitation bubble, thereby replacing the water in the surrounding area that refills the space vacated by the contraction of cavitation bubble.

As the *γ* continues to increase, the microjet of cavitation bubble near the air- free pit (Fig. 4(*b*_4_–*b*_7_)) resemble those near the rigid flat wall (Fig. 4(*a*_4_–*a*_7_)) in morphology. However, an air bubble was entrapped by pit, the cavitation bubble contraction causing the fluid motion, and pulls the air bubble out of the pit, forming a deformable boundary that weakened the constraining of the boundary. As a result, the cavitation bubble microjet velocity increases (v*≈14.9) (Fig. 4(*c*_4_–*c*_5_)). For *γ* > 1.6, The air bubble split into two parts: one inside the pit and the other outside the pit (as shown in Fig. 4(*c*_6_–*c*_7_)). Besides, the air bubbles had minimal effect on the cavitation bubble evolution, and the cavitation bubble collapse microjet morphology and velocity become similar to those observed near flat wall (Fig. 4(*a*_6_–*a*_7_)) and near pit without air bubble (Fig. 4(*b*_6_–*b*_7_)). It is noteworthy that the intense contraction of the cavitation bubble causes its internal temperature to rise, leading to luminescence phenomenon [54]. This luminescence interferes with the observation of the microjets.

**Fig. 5:**
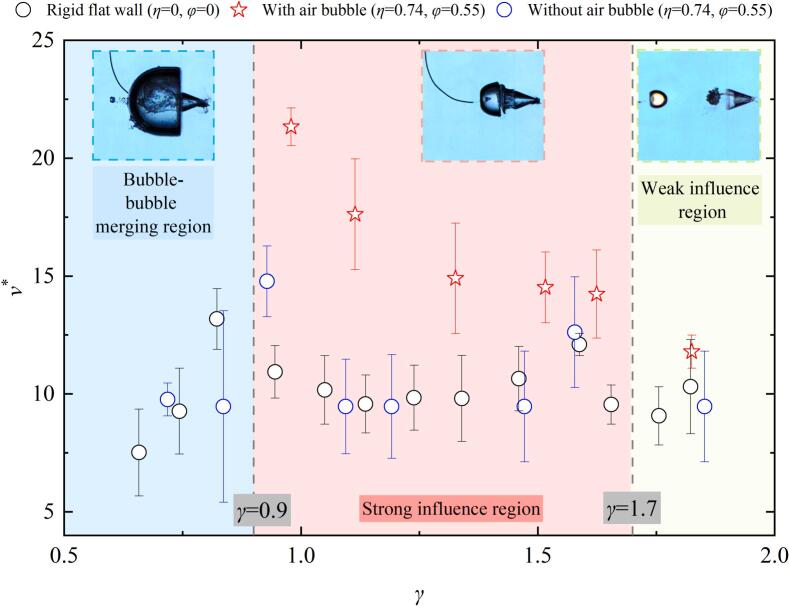
The relationship between the cavitation bubble microjet velocity and the cavitation bubble- wall distance.

Fig. 5 shows the variation of dimensionless microjet velocity (v∗) with γ. Near the rigid flat wall, the microjet velocity initially increases and then decreases. At γ ≈ 0.83, due to the more sufficient development time of the microjet within the cavitation bubble, v∗ reaches its maximum value of approximately 13.18, which is similar to the study of Philipp and Lauterborn (1998) [14]. However, when γ exceeds 1.5, the microjet velocity shows a slight increase. This can be attributed to the increased stand-off distance reduces wall confinement effect, significantly influence the contraction process and thus accelerating the bubble surface contraction velocity. Near a pit without an air bubble, where η = 0.74 and φ = 0.55, the variation of v∗ with γ follows a trend similar to that near the rigid flat wall, with a maximum v* of approximately 15.77 occurring around γ = 0.93. When pit contains an air bubble (η = 0.74, φ = 0.55, r_air_≈2mm), the variation of v∗ with γ is significantly different from that near the rigid wall or pit without air bubble. In this case, v∗ gradually decreases as γ increases. Within the range of 0.9 < γ < 1.7, the microjet velocity is 1.2 to 2.15 times higher than that near the rigid wall or pit without air bubble, indicating that small air bubbles within the pit strongly affect the microjet of cavitation bubble (as shown by the red region in Fig. 5). For γ > 1.7, the microjet velocity near the pit containing an air bubble becomes similar to that near the rigid flat wall, suggesting a negligible effect of the air bubble (shown by the green region in Fig. 5). For γ < 0.9, no clear microjet development is observed near the pit with an air bubble due to the merger of the cavitation bubble with the air bubble (as shown by the blue region in Fig. 5).

The above analysis suggests that when a pit exists on rigid wall, and small air bubble was trapped in pit, the evolution period of cavitation bubble decreases under same *γ* conditions, while the dimensionless microjet velocity shows an increasing trend. The reasons behind this phenomenon can be seen through the following factors: firstly, the pit’s geometric configuration elevates the dimensionless stand-off distance (defined as the ratio of bubble center to pit bottom separation to maximum bubble radius), thereby diminishing boundary effects on bubble dynamics. Secondly, ambient liquid rapidly occupies the volume vacated by the contracting bubble near the pit, enhancing surface collapse velocity and shortening the overall collapse duration.

### 3.3 Influence of small air bubble within pit on shockwave of cavitation bubble

Typically, cavitation erosion damage to boundaries is caused not only by microjet but also significantly caused by shockwave [55,56]. Luo et al [33] experimentally demonstrated that free-field cavitation bubbles interacting with neighboring air bubbles, the shockwave released by cavitation bubble collapse exhibits a stratification phenomenon, thereby achieving the goal of weakening the energy of shockwave. Kadivar et al [57] discovered that wall pits can focus the implosion shockwaves of cavitation bubbles, leading to secondary cavitation. Furthermore, the presence of a pit induces pressure fluctuations and stress concentration at the pit edges which exacerbates cavitation erosion [11]. Based on these findings, further research into the morphology and pressure of cavitation bubble implosion shockwaves influenced by pit with air bubble is crucial. Such research is essential for understanding the progression of cavitation erosion and for developing effective technologies aimed at preventing and controlling cavitation erosion. Therefore, in this chapter, we employ Schlieren imaging technique and transient pressure measurement systems [24,46] to synchronously record and analyze the influence of small air bubble within the pit on the morphology and pressure of cavitation bubble collapse.

**Fig. 6:**
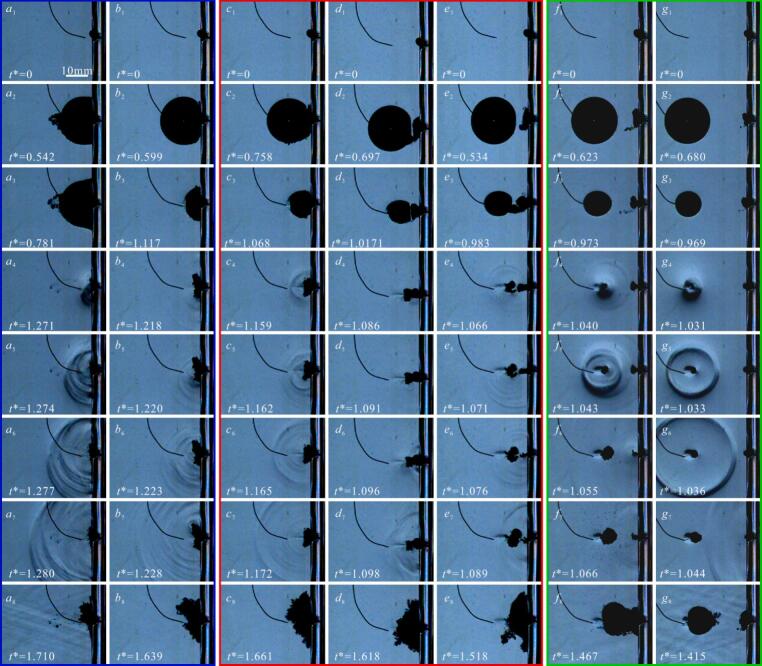
Effect of pit with air bubble on the morphology of cavitation bubble shockwave.

Fig. 6 is the cavitation bubble evolution process near a pit trapped a small air bubble and the morphology of the implosion shockwave under different γ conditions, captured using Schlieren imaging technique. Previous studies [33,45,58] have indicated that shockwaves are large localized pressure disturbances that propagate rapidly, with a short rise time and high amplitude (on the order of MPa). Based on this, the shockwave generated by cavitation bubble collapse is termed an ‘implosion shockwave’ in this study for two reasons: firstly, cavitation bubble collapse pressure test results show that the rise time of the shockwave is extremely short (4–30 μs). The pressure process of the collapse shockwave exhibits a sharp increase (rising to the MPa order) followed by a rapid decrease (dropping to zero or even negative pressure in the MPa order). Secondly, the shadowgraph images show that the shockwave is produced when the cavitation bubble contracts to its minimum volume, with a measured propagation speed of 1513 ± 58 m/s in high-speed images. It should be noted that the measured shockwave speed is influenced by the frame rate (180,000 fps) and spatial resolution (0.2273 mm/pixel) of the high-speed camera. If the propagation process of shockwave is not fully captured, the actual speed may be higher. Nevertheless, the measured values are slightly higher than the speed of sound in the liquid (approximately 1500 m/s), indicating a comparable order of magnitude. Therefore, the average shockwave speed from multiple experiments is used as the criterion for defining a ‘shockwave’ in this study. In Fig. 6a, (η = 0.37 φ = 0.55, r_air_≈1.5 mm, γ = 0.72), at t* = 0.542, the R_max_ = 10.31 mm, when t*=1.271, the cavitation bubble collapses and emits an implosion shockwave (Fig. 6(a_4_)). The shockwave exhibits a multi-layered structure and propagates outward (as shown in Fig. 6(a_5_–a_7_). When the time of cavitation bubble evolution (t*) is 1.710, the rebound of cavitation bubble is maximum (Fig. 6(a_8_)). In Fig. 6(b), with η = 0.37 φ = 0.55, r_air_≈1.5 mm, R_max_ = 10.95 mm and γ = 0.81. As the cavitation bubble collapses, the adjacent air bubble is drawn inward through the wall-adherent boundary region (Fig. 6(b_3_) and Fig. 2(c_3_)). At t* = 1.218, the cavitation bubble shockwave emitted is more diffuse compared to that in Fig. 6(a_4_), with fainter pressure traces in the Schlieren photography image. This diffuseness may be attributed to two primary factors: firstly, the development of a microjet leading to the formation of a toroidal bubble (see Supplementary Material Fig.S1). The asymmetric collapse of this toroidal structure generates multiple shockwaves with spatiotemporal variations, whose propagation and mutual superposition ultimately produce the diffuse shockwave pattern observed [33,59]. Secondly, the entrainment of air bubble into the cavitation bubble increases its internal content of non-condensable gas, thereby attenuates the shockwave [60]. The volume increases as the cavitation bubble rebounds to maximum, as the energy generated by the implosion shockwave during the first collapse is less than that in Fig. 6a, leaving more energy stored for rebound. In Fig. 6(c) (R_max_ = 11.1 mm, γ = 0.94). The number of shockwave stripes decreases, with only one single prominent stripe observed (Fig. 6(c_4_)). This may result from the air bubble buffering the microjet after it punctures the cavitation bubble surface. This prevents the formation of a distinct toroidal shape, leading to a more uniform and synchronous collapse. However, the air bubble absorbs some energy during buffering, which is later radiated as another shockwave at t* = 1.162. In Fig. 6(d), with R_max_ = 11.2 mm, γ = 1.10, the cavitation bubble’s expansion and contraction dynamics demonstrate consistent morphological features with those presented in Fig. 6(c_2_-c_3_), but the stretching length of air bubble along the pit depth is greater (approximately 6.51 mm). Besides, the air bubble buffering the microjet is more pronounced, and no distinct implosion shockwave is observed upon collapse to minimum volume (Fig. 6(d_4_)). Combining with the results in Fig. 5, the microjet velocity near the pit with an air bubble is highest, so we hypothesize that the cavitation bubble’s collapse energy is transformed into kinetic energy of the microjet, which is then cushioned by the air bubble. However, because the pit with small air bubble is subjected to microjet compression and constrained by the pit wall, itself undergoes an ‘implosion’, and generating a ‘shockwave’ (as shown in Fig. 6(d_6_-d_7_)). In Fig. 6(e), with R_max_ = 11.15, γ = 1.40, At t* = 0.983, the air bubble is entrained and coalesces with the cavitation bubble, followed by the collapse at t* = 1.066, releasing a weak implosion shockwave. However, the microjet is not buffered by the air bubble. The air bubble split along the pit surface, as a result of both the fluid flow generated by cavitation bubble collapse and adhesion to the pit wall. When the split air bubbles contract to their minimum volume, a clearly visible ‘implosion shockwave’ can once again be observed in the schlieren images (Fig. 6(e_6_–e_7_)). At γ = 1.8, the role of the air bubble in the cavitation bubble growth and collapse is significantly reduced. During contraction, a water hammer shockwave generated by the microjet impact on the surface of bubble (Fig. 6(f_4_)). Subsequently, the cavitation bubble collapse and generates an implosion shockwave at t* = 1.043. At the same time, the cavitation bubble’s effect on the air bubble is likewise reduced, a faint shockwave is also observed in the schlieren images when the air bubble contracts to its minimum volume (as shown in Fig. 6(f_6_–f_7_)). In Fig. 6(g), R_max_ = 11.14 mm, γ = 2.45, the perturbations induced by the pit and the air bubble on the cavitation bubble are minimal. The cavitation bubble collapses symmetrically, and emitting a shockwave propagates outward in a 'regular spherical' pattern (Fig. 6(g_4_–g_6_)). No distinct ‘implosion shockwave’ is observed during the air bubble’s collapse.

**Fig. 7:**
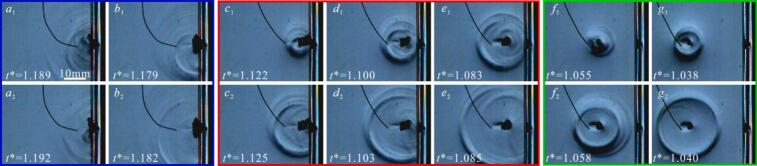
Effect of pit without air bubble on the morphology of cavitation bubble shockwave.

Fig. 7 shows the morphology of the cavitation bubble implosion shockwave near a pit (*η* = 0.37, *φ* = 0.55) without air bubble for *γ* corresponding to those in Fig. 6. The results reveal that the shockwave morphology of the cavitation bubble in Fig. 7(a) and (b) is also multi-layered, similar to that observed in Fig. 6(*a*_4_) and Fig. 6 (*b*_4_). This similarity occurs because the non-uniform thickness of the toroidal cavitation bubble leads to spatial and temporal differences during its collapse. In Fig. 7(*c-e*) (*γ* = 0.97, 1.12, and 1.39 respectively), experimental results show that cavitation bubbles collapsing near without air bubble in pits, exhibit a more distinct implosion shockwave of cavitation bubble with a multi-layered structure compared to cases with an air bubble (such as Fig. 6(*c–e*)). This suggests that within this range of *γ*, the air bubble can significantly attenuate cavitation bubble collapse intensity. However, for *γ* > 1.8, the morphology of the cavitation bubble shown in Fig. 7(*f–g*) becomes similar to that in Fig. 6(*f–g*), indicating that the collapse characteristics show little sensitivity to air bubble presence under the implemented conditions.

**Fig. 8:**
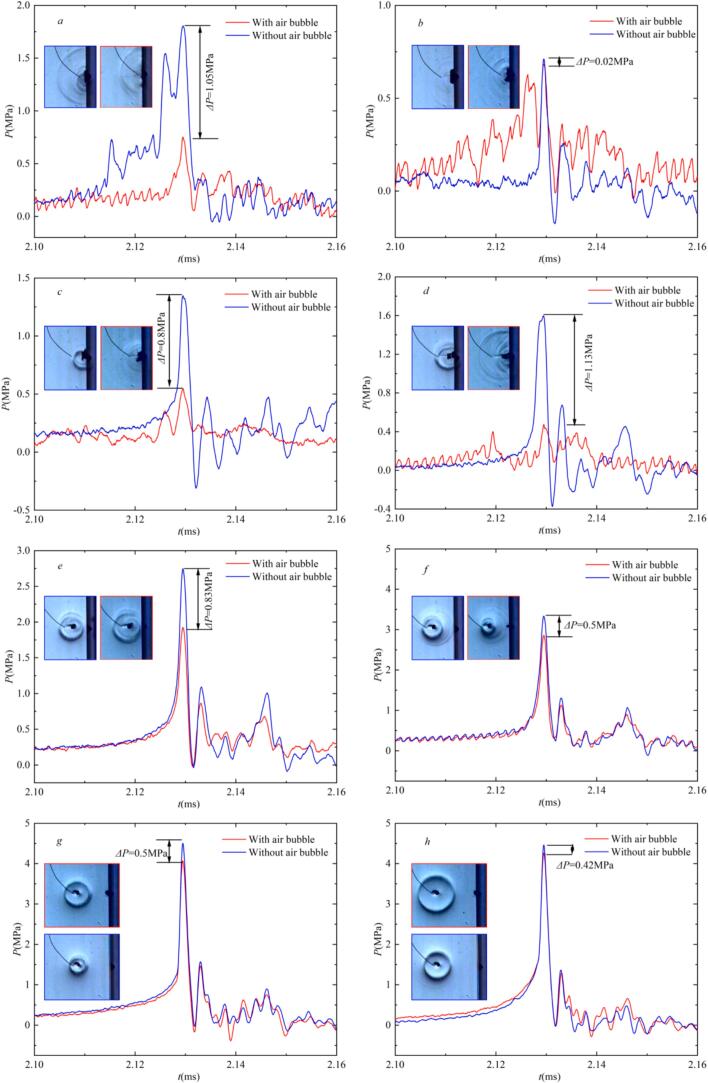
Pressure signals of cavitation bubble shockwave under the influence of air bubble within pit.

From Fig. 6 and Fig. 7, it is evident that for a given *γ*, the small air bubble within the pit significantly influences the cavitation bubble implosion shockwave. To quantify the shockwave pressure, a high-frequency pressure sensor was installed along the pit’s central axis in the cavitation bubble’s centroid plane, maintaining a dimensionless distance of 3 between the sensor chip and bubble center. The corresponding cavitation bubble implosion shockwave pressure signals are shown in Fig. 8.

Fig. 8(a) compares the pressure evolution during shockwave propagation for cases with and without air bubble at η = 0.37, φ = 0.55, and γ = 0.69. When an air bubble (r_air_≈1.5 mm) was trapped by the pit, the cavitation bubble implosion shockwave pressure is approximately 0.75 MPa. However, near the pit without an air bubble, the cavitation bubble implosion shockwave not only presents multiple signal peaks but also the maximum peak pressure is about 1.80 MPa. The air bubble reduces the cavitation bubble implosion shockwave pressure by about 58 %. This attenuation is attributed to the entrainment of non-condensable gas, which absorbs collapse energy [33]. In Fig. 8(b), γ = 0.84, the cavitation bubble implosion shockwave pressure near with and without air bubbles in the pit were relatively small, at 0.69 MPa and 0.71 MPa, respectively. At γ = 0.93, the damping of air bubble in pit on the collapse microjet of cavitation bubble (Fig. 6c) reduced the peak implosion pressure by approximately 0.8 MPa compared to the implosion shockwave pressure near the pit without air bubble (Fig. 8(c)). The pressure signal in Fig. 8(d) (γ = 1.10) shows significant attenuation by the air bubble (as the red curve in Fig. 8(d)), with the peak pressure being only about 0.47 MPa. However, in the absence of the air bubble in pit, the reduced boundary constraint resulted in a more concentrated peak pressure of about 1.6 MPa. As the dimensionless distance γ increase further, the effect of small air bubble in pit on the cavitation bubble diminishes. At this time, the pressure progress of implosion shockwave shows only a single peak. Furthermore, the time span of the shockwave pressure process with and without air bubbles is almost the same (as shown in Fig. 8(e–h)). When air bubbles are present, the cavitation bubble implosion shockwave pressure of is smaller by 0.83, 0.5, 0.5, and 0.42 MPa, respectively, compared to when there are no air bubbles. This demonstrates that the air bubble provides attenuation of cavitation bubble shockwave intensity to some certain conditions. However, its influence on the implosion shockwave intensity gradually decreases as γ increases. Based on experimental observations, the reduction in shockwave pressure maximum caused by the air bubble can be broadly divided into a strong influence region and a weak influence region based on the values of γ. Furthermore, a detailed discussion of air bubble influence zones and their impact on shockwave peak pressures of cavitation bubble follows.

**Fig. 9:**
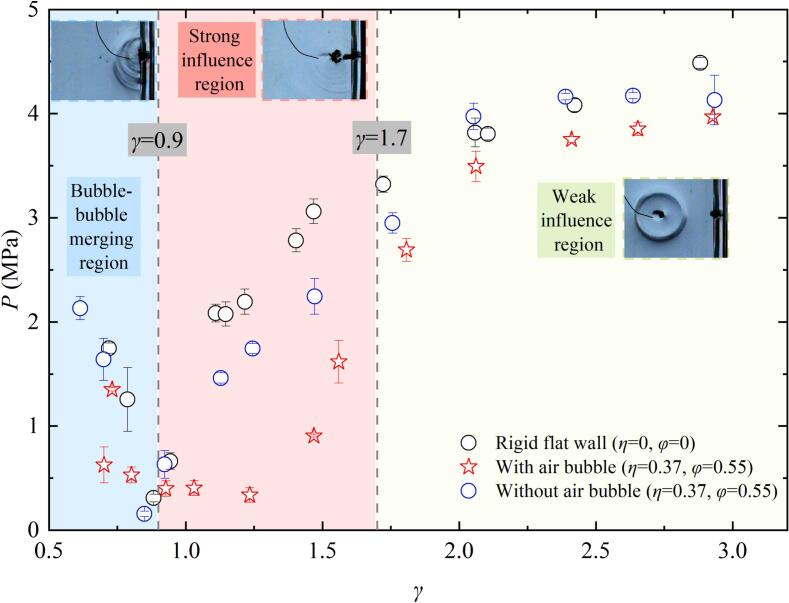
Relationship between the cavitation bubble- wall distance and the cavitation bubble shockwave pressure.

Fig. 9 compares the peak pressures of implosion shockwaves generated by cavitation bubble collapse under three conditions: (1) near a rigid flat wall, (2) near a pit without an air bubble, and (3) near a pit containing an air bubble. as *γ* increases, the peak pressure of cavitation bubble collapse adjacent to a rigid boundary and the pit without air bubbles initially decreases, then increases, and eventually stabilizes. However, the presence of a small air bubble in the pit, the shockwave intensity is initially attenuated, then stabilizes, followed by a sharp increase, and eventually stabilizes again. The minimum shockwave peak pressure occurs at *γ≈*0.83. Below this threshold (*γ* < 0.83), the implosion pressure shows negligible variation between rigid flat wall and air-free pit configurations. However, due to the cavitation bubble combined with the small air bubbles in the pit, which lead to the implosion shockwave pressure of cavitation bubble drastically decreases. This region is defined as bubble–bubble merging region (as the blue region of Fig. 9). For 0.9 < *γ* < 1.7, the interaction between the air bubble and the cavitation bubble becomes more pronounced, as evidenced by Fig. 2, Fig. 6, Fig. 7. Significant differences in collapse shockwave pressure are observed between cases with and without an air bubble. Therefore, this region is defined as the Strong influence region. At *γ*≈1.7, the presence of air bubbles in the pit reduced the peak pressure of the cavitation bubble collapse shockwave by approximately 28 % compared to that near a rigid flat wall, and by approximately 10 % compared to that near a pit without air bubbles. At *γ*≈1.25, the closer standoff distance enhanced the interaction between the air bubble and the cavitation bubble, resulting in more pronounced pressure attenuation. The cavitation bubble collapse shockwave pressure was reduced by approximately 85 % compared to the case near a rigid flat wall, and by nearly 80 % compared to that near a pit without air bubbles. Additionally, as *γ* increases, the pressure peak attenuation caused by air bubbles becomes less pronounced. For *γ* above approximately 1.7, as observed in Fig. 6 and Fig. 7, the shape of the shockwave remains similar, regardless of the presence or absence of air bubbles. Additionally, there is no significant difference in the cavitation bubble collapse shockwave pressure (Fig. 9) or microjet velocity (Fig. 5). Hence, this region is classified as the Weak influence region (as shown in the green area of Fig. 9). Especially for *γ* > 2, the pit’s effect on cavitation bubble implosion shockwaves becomes negligible, and the ability of small air bubbles in the pit to reduce the shockwave peak pressure weakens.

**Fig. 10:**
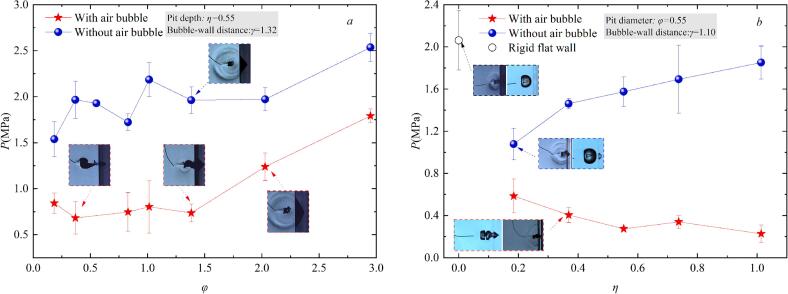
Cavitation bubble collapse shockwave peak pressure near different pits.

This study systematically examines how pit geometry (characterized by diameter *φ* and depth *η*) modulates cavitation-induced shockwaves within the constrained dimensionless stand-off range (1 ≤ *γ* ≤ 1.3). As shown in Fig. 10(a), when *γ* is constant (*γ* ≈ 1.32) and no small air bubble is present in the pit, the maximum shockwave pressure generated during cavitation bubble collapse increases by approximately 40 % overall with increasing *φ*. This occurs because a larger *φ* reduces the constraint imposed by the rigid wall on the cavitation bubble, leading to a more symmetric collapse and increase in the peak pressure of the cavitation bubble collapse. However, for *φ* < 1.5, there is a slight fluctuation in the cavitation bubble collapse pressure. This instability may be caused by asymmetric collapse of the toroidal cavitation bubble induced by smaller pit diameters. When a small air bubble (*r*_air_ = 1.5–2.5 mm) are present in pits with different diameters *φ*, the cavitation bubble implosion shockwave pressure decreases compared to pits without air bubble. This is because a larger pit diameter (*φ*) provides a greater volume to accommodate the expanded air bubble. As a result, the elongated air bubble is less likely to merge with the cavitation bubble (as shown in Fig. 10a). Consequently, the peak pressure of the implosion shockwave initially stabilizes and then increases with increasing *φ*, with the peak pressure difference being about 1.11 MPa. Fig. 10(b) indicates the effect of pit depth (*η*) on the cavitation bubble implosion shockwave pressure. The results indicate that in pits without air bubble, the cavitation bubble implosion shockwave pressure increases as *η* increases, with an increase of about 42 %. However, it is still lower than the shockwave peak pressure of cavitation bubble near the rigid flat wall. The underlying reasons for this difference can be explained as follows: at *γ*≈1.1, after the microjet punctures the cavitation bubble surface along the wall normal direction, the resulting toroidal bubble collapses more symmetrically, leading to a higher shockwave peak pressure. In contrast, the presence of small pits on the wall introduces asymmetry in the toroidal bubble’s spatial morphology, which causes the shockwave propagation more disperse, thereby reducing the peak pressure. As the pit depth increases, the space between the cavitation bubble and the wall increases, weakening the wall’s confinement effect and allowing the cavitation bubble collapse to become more violent. For small air bubble is present in the pit, the implosion shockwave pressure gradually decreases with increasing pit depth (*η*), reaching a maximum attenuation amplitude of 60 %. The possible reason is that increased pit depth results in a narrower and deeper geometry. Under the influence of the cavitation bubble, the air bubble in the pit becomes more elongated along the wall-normal direction, which delays the interaction between the air bubble and the cavitation bubble, ultimately reducing the shockwave pressure.

The increase of pit diameter and depth result to the increase of cavitation bubble collapse pressure may be attributed to two reasons: firstly, as the pit diameter and depth increase, increased standoff distance from the pit boundary results in reduced confinement effects on the collapse of cavitation bubble. Secondly, the increase in pit depth and diameter produces an increase in the water volume within the pit. The water within the pit refills the space released as the cavitation bubble contracts, resulting in a more uniform and intense implosion. When the pit contains small air bubbles, the shockwave pressure decreases. As noted by Luo et al. [33], when the air bubbles maintain separation from the cavitation bubble, the multilayer shockwave of cavitation bubble forms, thereby reducing the shockwave intensity. We speculate that this pressure reduction relates to alterations in the cavitation bubble collapse morphology. During the cavitation bubble evolution process, the contraction of the cavitation bubble drives the surrounding liquid movement, which further causes the air bubble within the pit to undergo a compression and rebound. Additionally, the oscillation of the air bubble itself also transfers energy through the liquid medium, thereby exerting an attractive effect on the cavitation bubble, which in turn affects the morphology of the cavitation bubble. When the *η* increases, the reduction in cavitation bubble collapse pressure may be due to the fact that with the pit diameter remaining constant, an increase in depth makes the internal space of the pit more elongated. In this case, the combined effects of compression (during expansion) and fluid-driven tension (during contraction) promote axial elongation of the air bubble. Such deformation can lead to cavitation bubble-air bubble coalescence or microjet damping by air bubble, ultimately reducing collapse aggressiveness.

## 4 Effect of cavitation bubble collapse on small air bubble in pit

In Chapter 3, how pit-confined air bubbles alter cavitation collapse dynamics was analyzed through controlled experiments. It was also found that under constant *γ* conditions, small air bubbles within the pit undergo violent collapse induced by the cavitation bubble. In this section, we will further analyze the ‘implosion shockwave’ phenomenon of small air bubbles in the pit under the effect of cavitation bubbles and the critical conditions for its occurrence. The shockwave generated by the small air bubble to its minimum volume is defined as an 'implosion shockwave' for two main reasons: firstly, the shockwave originates from the collapse of the small air bubble within the pit. Secondly, high-speed imaging reveals that the shape and propagation speed of the ‘implosion shockwave’ (1534 ± 39 m/s) are similar to those of the implosion shockwave generated by cavitation bubble collapse (1513 ± 58 m/s), with both of speeds reaches the order of the speed of sound in the liquid (∼1500 m/s).

### 4.1 The ‘implosion shockwave’ phenomenon of small air bubble within pit

**Fig. 11:**
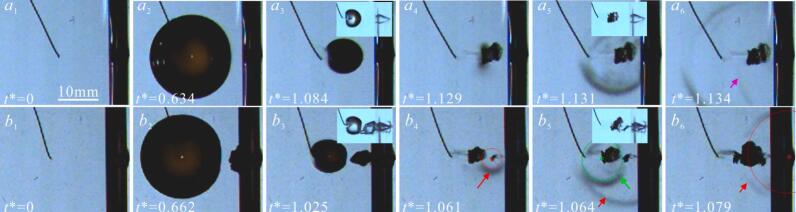
‘Implosion shockwave’ phenomenon of air bubble in pit induced by cavitation bubble.

Fig. 11 shows ‘implosion shockwave’ phenomenon of an air bubble in pit induced by cavitation bubble. Fig. 11(a) is the situation without small air bubbles in the pit. The shape of cavitation bubble is almost spherical in Fig. 11(*a*_2_). A conical microjet forms during the cavitation bubble contraction (Fig. 11(*a*_3_)). At t* = 1.129, the cavitation bubble collapse (Fig. 11(*a*_4_)). The shockwave radiated by cavitation bubble then forms a ring and expands outward (Fig. 11(*a*_5_)), as the cavitation bubble collapse shockwave impacts the wall, it undergoes reflection, resulting in a reflected shockwave (as shown in Fig. 11a6*).* When a small air bubble is present in the pit, the development of cavitation bubble is similar to Fig. 11(a). However, at the stage of cavitation bubble expansion, the air bubble expands, rebounds, and overflows from the pit (Fig. 11(*b*_2_)). As shown in Fig. 11(*b*_3_), the air bubble within the pit is further elongated by the contracting cavitation bubble. At t* = 1.061, the collapse of the air bubble portion outside the pit generates an ‘implosion shockwave’, as indicate by the red arrow in Fig. 11(*b*_4_). Notably, the cavitation bubble has not yet reached its minimum volume. By employing center-positioning method, which identifies the radiation source by tracking the center of the shockwave front, it is confirmed that the observed shockwave originates from the collapse of air bubble outside the pit. At t* = 1.064, the shockwave generated by the cavitation bubble collapse begins to radiate outward (as shown by the green arrow in Fig. 11(*b*_5_)). However, the part of the air bubble inside the pit has not yet reached its minimum volume. By t* = 1.079, the air bubble inside the pit collapses. Meantime, a ‘shockwave’ propagating outward from the bottom of the pit was observed (as the red arrow in Fig. 11(*b*_6_). Based on the shockwave propagation velocity in water (≈1500 m/s), the round-trip travel time to the pit bottom (*η* = 0.55) would be only about 8 μs—significantly shorter than the 33 μs interval between Fig.11b5 and Fig.11b6. This confirms that the ‘shockwave’ originates from the air bubble collapse rather than a wall reflection.

**Fig. 12:**
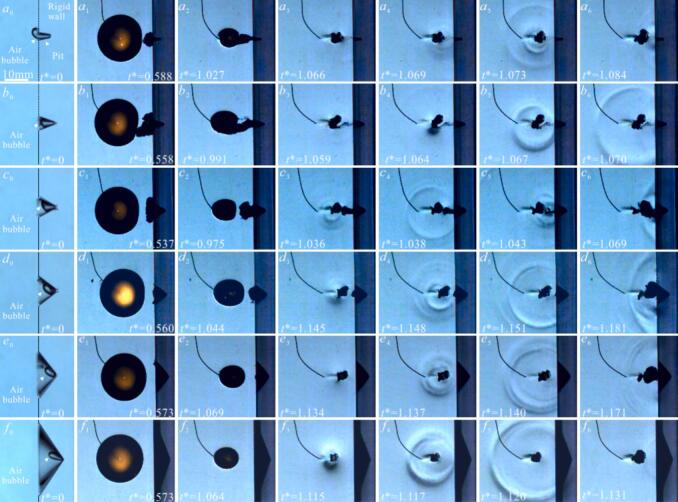
Influence of pit diameter on the morphology of ‘implosion shockwave’ of air bubble in pit.

Section 3.3 revealed that the generation of cavitation bubble shockwaves depends on *γ*, and that pit dimensions also affect the cavitation bubble implosion shockwave. However, does the generation of the air bubble ‘implosion shockwave’ also depend on the pit dimensions? We further analyzed the variation of the air bubbles in pits of different sizes under the influence of cavitation bubbles, as shown in Fig. 12. Similarly, by combining methods such as center positioning method and shockwave timing analysis, we distinguished between the ‘implosion shockwave’ originating from the cavitation bubble and those from the air bubble. The initial morphologies of the air bubbles within pits of different size are shown in Fig. 12a0–f0. Since Fig. 12 primarily analyzes the 'implosion shockwave' phenomenon of small air bubble inside pit of different sizes, the evolution characteristics of air bubble in pit under the influence of cavitation bubble can be found in the Supplementary Material, Fig. S3.

In Fig. 12(a), *r*_air_≈1.5 mm, *η* = 0.55, *φ* = 0.18, *γ*≈1.3, *R*_max_ = 11.21 mm. Compared to Fig. 11(b), the difference lies in the stage at which the contraction of cavitation bubble. At t* = 1.027, the collapsing cavitation bubble generates attractive to the air bubble, but the air bubble does not completely merge (Fig. 12(*a*_2_)). Subsequently, the unmerged residual portion of the air bubble undergoes compressive collapse, and generates a weak ‘implosion shockwave,’ as shown in Fig. 12(*a*_3_–*a*_4_). At t* = 1.073, the cavitation bubble collapse and produces a circular ‘implosion shockwave’ (as shown in Fig. 12(*a*_5_)). In Fig.12a6, Residual air bubbles remaining in the pit subsequently emit another 'implosion shockwave' upon contracting to their minimum volume. In Fig. 12(b), *R*_max_ = 11.29 mm, *φ* = 0.37, *r*_air_≈2mm, *η* = 0.55, *γ*≈1.3. During the cavitation bubble contracts stage, the air bubble is pulled out of the pit (Fig. 12(*b*_2_)), and the air bubbles are almost completely merges with the cavitation bubble. The collapse generates an axisymmetric shockwave (Fig. 12(*b*_3_-*b*_8_)), and there almost no noticeable air bubble ‘shockwave’ after the air bubble being fully engulfed by the cavitation bubble. The reason for this phenomenon is that the pit diameter *φ* is small, leading to a smaller contact area between the air bubble and the pit wall. The surrounding fluid flow generated during bubble contraction enhances air bubble dislodgement from the pit wall, resulting in full merger with the cavitation bubble. In Fig. 12(c), *φ* = 0.55, the cavitation bubble contraction extracts the air bubble from the pit, but the bubbles remain independent and do not merge (Fig. 12(*c*_2_)). This is attributed to the enlarged pit dimensions increase the air bubble’s contact area with the pit base, thereby enhancing surface adhesion forces that constrains the air bubble full coalescence during cavitation events. At t*=1.036, the cavitation bubble radiates an implosion shockwave (Fig. 12(*c*_3_-*c*_4_)). The air bubble collapses later (t* = 1.043), generating a more pronounced implosion shockwave than in cases Fig. 12(*a-b*). When the portion of the air bubble confined within the pit contracts to its minimum radius, it radiates a clearly observable ‘implosion shockwave’ that propagates toward the outside of the pit (Fig. 12(*c*_6_)). In Fig. 12(d), *R*_max_ = 10.9 mm, *φ* = 0.83, the increased pit volume accommodates the expanded air bubble. Despite being subjected to the attraction and compression induced by cavitation bubble, the enlarged air bubble maintains structural integrity without splitting (Fig. 12(*d*_2_)). The collapse phase generates dual shockwave components − a water hammer from microjet impact and spherical implosion shockwaves − which interact to form the stratified morphology visible in Fig. 12(*d*_4_). Because the air bubble does not split along the pit surface, only a single ‘implosion shockwave’ originates from the pit bottom upon its collapse (Fig. 12(*d*_6_)). Figs. 12(e) and (f) show the air bubble ‘implosion shockwave’ variation process when the pit diameter *φ* is 1.38 and 2.95, respectively. As the increases of pit diameter, the wall’s influence on the cavitation bubble diminishes, leading to the cavitation bubble more uniform contraction. Consequently, the cavitation bubble implosion shockwave appears as nearly a regular circular in the Schlieren images. The pit volume increases as the pit diameter *φ* increases, allowing the air bubble to deform and expand in a larger space inside the pit, preventing excessive compression of the air bubble. Meanwhile the air bubble contraction process slows down, accompanied by ‘implosion shockwave’ of air bubble significantly weakens (as the Fig. 12(*e*_6_), Fig. 12(*f*_6_)).

The generation of ‘implosion shockwave’ from air bubbles within small pits under small *γ* conditions can be explained from two aspects: On the one hand, the confined space of a small pit subjects the air bubble to intense compression, causing pressure from the surrounding liquid to be converted and stored within the air bubble. On the other hand, the shockwave generated by the cavitation bubble collapse elevates the ambient liquid pressure, creating a transient pressure differential that drives rapid compression of the air bubble. The strongly compressed air bubble subsequently releases its stored energy in the form of an ‘implosion shockwave’.

### 4.2 The critical condition ‘implosion shockwave’ of small air bubble within pit

**Fig. 13:**
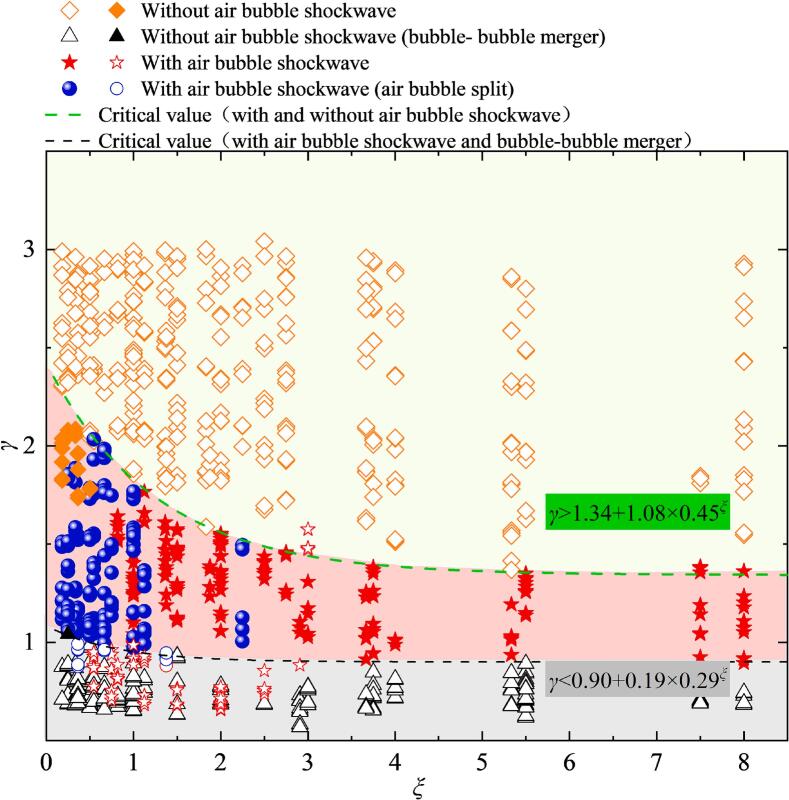
Critical conditions for the ‘implosion shockwave’ of air bubble in pit induced by cavitation bubble collapse.

From Fig. 6 and Fig. 12, it can be observed that when the dimensionless distance of *γ* and pit spatial size *ξ* (*ξ = W/H*) under certain conditions, air bubble within the pit will generate an ‘implosion shockwave’ phenomenon under the action of cavitation bubble. Therefore, Fig. 13 analyzes the critical conditions for generation of ‘implosion shockwave’ under varying *γ* and *ξ*. In Fig. 13, the symbol  represents cases where the air bubble collapse does not exhibit ‘implosion shockwave’ phenomenon, indicates that the air bubble merges with the cavitation bubble, and thus no obvious ‘implosion shockwave’ of air bubble.  denotes the case where the air bubble generates a single ‘implosion shockwave’.  represents the cases where the air bubble splits, which generates two ‘implosion shockwave’. The results revealed that as *ξ* increases, the critical *γ* required for air bubble ‘implosion shockwave’ formation decreases. Additionally, when the pit size is narrow and deep (*ξ* < 2.5), the air bubble is more likely to split into two parts, located inside and outside the pit.

Based on the experimental findings, the critical dimensionless distance γ* for the collapse of an air bubble in the pit that emits an ‘implosion shockwave’ follows an exponential decay pattern as *ξ* increases, a regression analysis [61] of the relationship between γ* and *ξ* was performed, specifically described by:

(4) $$\gamma *=A+B\times C^{\xi }$$

The coefficients *A*, *B*, and *C* for the critical curve of small air bubbles generating the 'implosion shockwave' within the pit are provided in Table 2.

**Table 2:** Critical curve coefficients.

	*A*	*B*	*C*
Critical curve 1	1.34	1.08	0.45
Critical curve 2	0.90	0.19	0.29

Specifically, in case of *γ* < 0.90 + 0.19 × 0.29*^ξ^* (Critical curve 1), the cavitation bubble and the air bubble are likely to merge, making it difficult to distinguish the air bubble ‘implosion shockwave’ from the cavitation bubble implosion shockwave. However, in some experiments, the air bubble ‘implosion shockwave’ can still be identified, potentially due to incomplete merging of the air bubble with the cavitation bubble (for example, for *ξ* < 2.5, the air bubble may split along the pit surface, and the air bubble remaining the pit can generate an ‘implosion shockwave’). For *γ* > 1.34 + 1.08 × 0.45*^ξ^* (Critical curve 2), the air bubble to no longer undergoes violent contraction, and thus no visible ‘implosion shockwave’ of air bubble is observed in the Schlieren images. These criteria (Equation 4) are fitted from the experimental data and are specifically applicable to small pits (*φ* < 3, *η* < 1.1) with a limited range of bubble size ratios (*r*_air_/*R*_max_ = 0.2–0.25). Their generalizability to scenarios involving different water properties, pit shapes, bubble size ratios, or interactions near flexible or rigid flat walls requires further systematic investigation.

Further analysis of the coefficients in equation (4) reveals that coefficient *A* is related to the critical condition for whether small air bubbles in the pit emit an ‘implosion shockwave’ with the upper and lower limits approximately 1.34 and 0.9, respectively. Coefficient *B* represents the coefficient of the exponential function, while coefficient *C* corresponds to the rate of change in the exponential function, it likely reflecting the sensitivity of γ* to *ξ*. Since the expansion and collapse of air bubble in pit under the cavitation bubble influenced by *γ* and *ξ*, the ratio of the maximum bubble radii of the air bubble and the cavitation bubble (*R*_air_/*R*_max_) was found to vary within a range of approximately 0.2 to 0.42. It is noteworthy that the fitted values for C also fall within this range. Based on this observation, we hypothesize a potential correlation between Coefficient *C* and the size ratio *R*_air_/*R*_max_. However, a definitive quantitative relationship between them requires further experimental investigation.

It should be noted that the above findings are a summary based on the experimental data under current conditions. To accurately reveal the coefficients in the critical curve, further in-depth studies are needed, focusing on the interaction between air bubbles, cavitation bubbles, and pits at different scales.

## 5 Conclusions

To study the interaction between air bubbles or gas nuclei entrapped within pits on water flow boundaries and cavitation bubbles within cavitation clouds or bubble clusters. A controlled cavitation bubble was induced by underwater Corona Discharge. A high-speed photography system and high-frequency pressure measurement system were employed to study the interaction between air bubbles within pits and cavitation bubbles. The main conclusions are as follows:

(1) Under certain conditions, the dynamics process of cavitation bubble is significantly influenced by a small air bubble in pits. Specifically, for a given pit spatial size(*ξ*) and air bubble size, as the dimensionless distance (*γ*) from the cavitation bubble to the wall increases, the evolution period of cavitation bubble gradually shortens. The implosion shockwave pressure of cavitation bubble initially decreases, then increases, and finally tends to stabilizes. Based on experimental observations, it was found that for *γ* is less than approximately 0.9, the air bubble is entrained by the cavitation bubble. In the range of *γ* between 0.9 and 1.7, the presence of air bubbles shortens the cavitation bubble evolution period, attenuates the implosion shockwave pressure, but increases the dimensionless microjet velocity compared to the situation without an air bubble in pits.
(2) By combining high-speed photography with the Schlieren technique, an ‘implosion shockwave’ phenomenon generated by the collapse of air bubbles within the pit under the influence of a nearby cavitation bubble was observed. This indicates that beyond attenuating the pressure of cavitation bubble collapse shockwaves, air bubbles entrapped in pits may also transform into a new source of ‘shockwave’ under certain conditions. The critical conditions for the air bubble generate ‘implosion shockwave’ were summarized as follows: quantitative analysis reveals that as the pit spatial size *ξ* increases, the critical dimensionless distance (γ*) between the surface of the pit and the center of the cavitation bubble follows an exponential decay trend (γ* = *A* + *B × C^ξ^*). The coefficient *C* represents the rate of change of the exponential function, which is likely related to the ratio of the maximum radii of the air bubble and the cavitation bubble (*R*_air_/*R*_max_). Specifically, as the *γ* is less than 1.34 + 1.08 × 0.45*^ξ^*, the small air bubbles in the pits exhibit the ‘implosion shockwave’ phenomenon. in case of *γ* < 0.90 + 0.19 × 0.29*^ξ^*, the air bubble in the pit merges with the cavitation bubble, making it difficult to distinguish whether ‘cavitation’ is occurring in the air bubbles within pit.

These innovative findings from the above experiments provide a foundation for theoretical studies on how the smoothness of water flow boundaries affects cavitation and potential erosion of these boundaries. Additionally, these findings offer references for assessing and controlling cavitation intensity in pitted regions, as well as for designing aeration strategies to mitigate erosion.

## 6 Originality statement

We assure that the contents of this contribution are original, and this paper has not been submitted to any other journal for publication, or not published before elsewhere, and this article contains no libelous or unlawful statements, and does not infringe on the rights of others.

## CRediT authorship contribution statement

**Jie Li:** Writing – original draft, Visualization, Investigation, Formal analysis, Data curation. **Siyu Chen:** Investigation. **Jing Luo:** Writing – review & editing, Supervision, Methodology, Funding acquisition. **Weilin Xu:** Supervision, Methodology, Funding acquisition, Conceptualization. **Jiguo Tang:** Investigation. **Tong Qu:** Investigation, Formal analysis, Data curation.

## Declaration of competing interest

The authors declare that they have no known competing financial interests or personal relationships that could have appeared to influence the work reported in this paper.
