# Supplementary material for: Shockwaves from air bubbles within pits induced by nearby cavitation bubbles
Source: Ultrason Sonochem. 2025 Oct 3;122:107602. doi: 10.1016/j.ultsonch.2025.107602 (PMC12547827; doi:10.1016/j.ultsonch.2025.107602)
Supplement: Supplementary Data 1 [file mmc1.docx]

**Shockwaves from Air Bubbles within Pits Induced by nearby Cavitation Bubbles**

Jie Li,^1^ Siyu Chen,^2^ Jing Luo,^1,^ ^[[1]](#footnote-1)^ Weilin Xu,^1^ Jiguo Tang,^1^ Tong Qu^1^

^1^ *State Key Laboratory of Hydraulics and Mountain River Engineering, Sichuan University, Chengdu 610065 China*

^2^ *Hydraulics Department, Changjiang River Scientific Research Institute, Wuhan 430010 China*

# S.1. Toroidal cavitation bubble morphology

Fig. S.1 shows the toroidal cavitation bubble morphology formed when the microjet within the cavitation bubble impacts the opposite bubble surface, the cavitation bubble adopts a ‘doughnut’ shape and releases a water hammer shockwave (Fig. S1 *b*_3_). Subsequently, as the toroidal bubble contracts to its minimum volume, it emits an implosion shockwave (Fig. S1 *b*_4_).


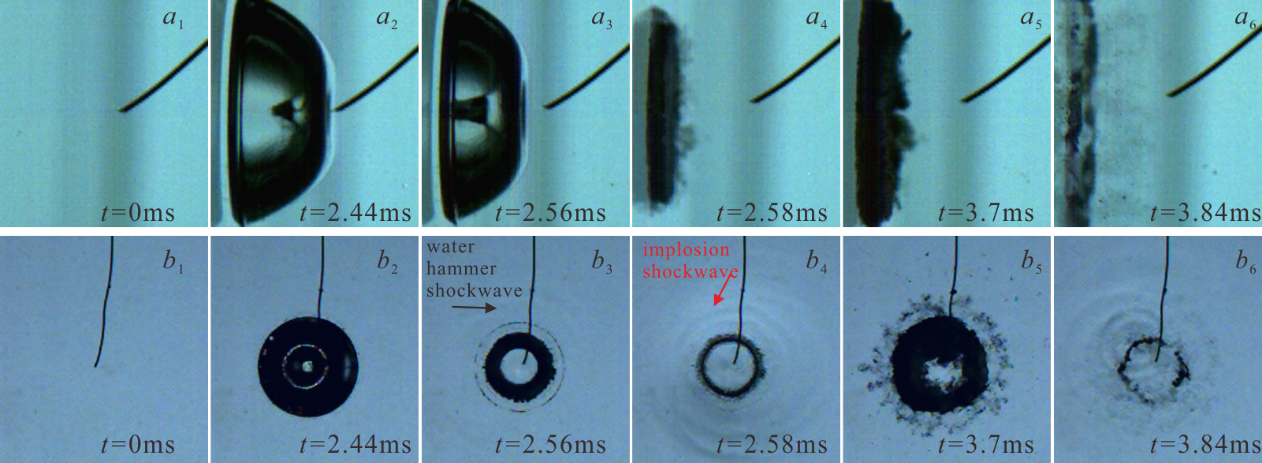


Fig. S.1 The morphology of the toroidal bubble formed after the microjet punctures the cavitation bubble (a)Side view (b)Front view

# S.2. Influence of saturated vapor pressure on the variation trend of microjet velocity

Fig. S.2 shows the variation in microjet velocity for *P*ᵥ = 0 kPa and *P*ᵥ = 20 kPa. It can be observed that although different values of *P*ᵥ lead to certain differences in the specific numerical results of *v**（*v**=*v*/(Δp/ρ) ^0.5^）, they do not significantly affect the overall trend of microjet velocity. Furthermore, the choice of *P*ᵥ does not alter the relative magnitudes of *v** under conditions involving a rigid wall, the presence of an air bubble in pits, or the absence of air bubble in pits.


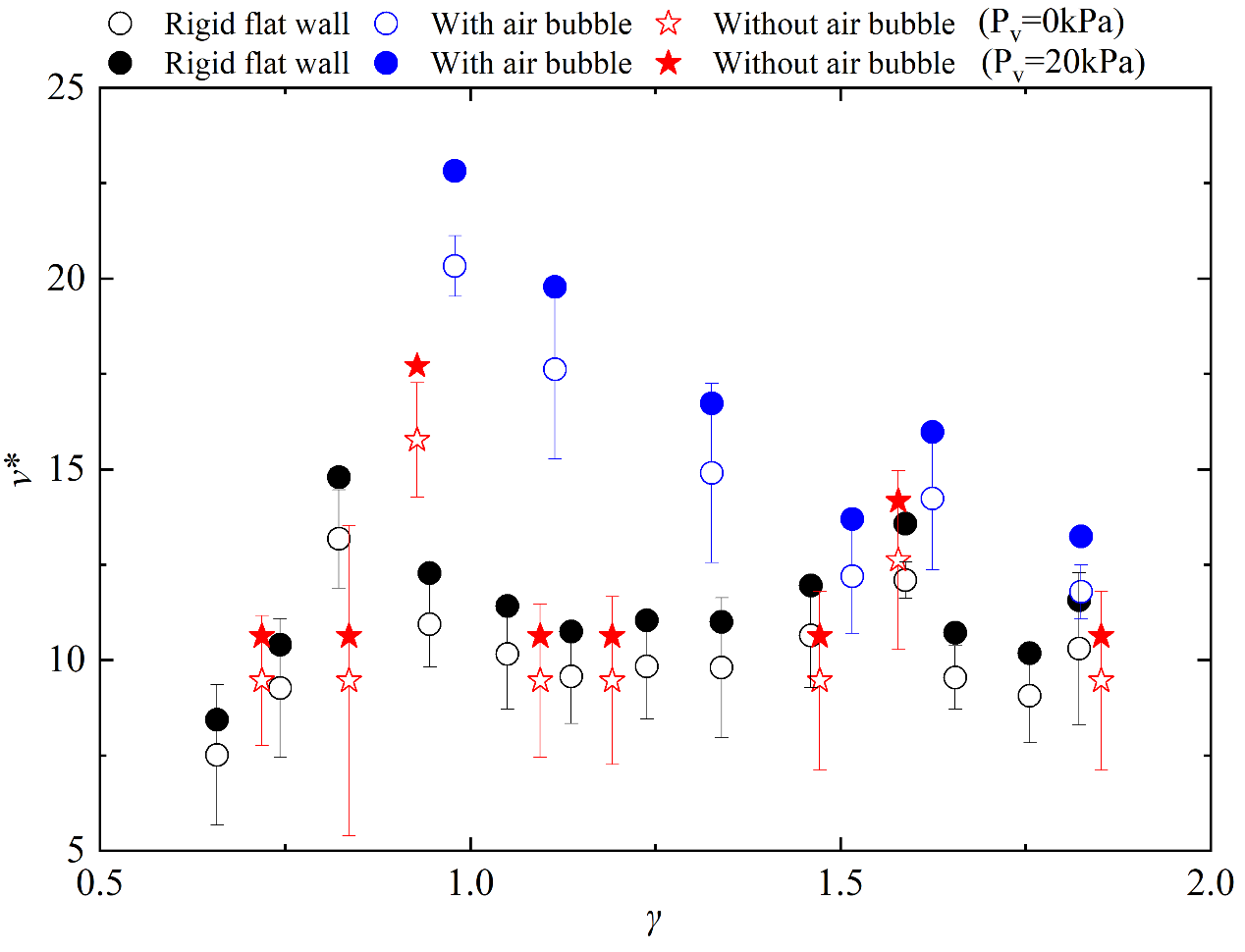


Fig. S.2: Influence of saturated vapor pressure on the variation trend of microjet velocity.

# S.3. Influence of pit diameter on the morphology of air bubble in pit

In Fig. S3(*a*) (*R*_max_ = 11.05 mm), the contraction of the cavitation bubble attracted and subsequently merged with the small air bubble in the pit (Figs. S3 *a*_2_-*a*_4_). For a pit with *φ* = 0.37 (Fig. S3(*b*)), the cavitation bubble contraction causes the small air bubble to elongate and split into two parts along the pit surface: one inside and one outside the pit (Fig. S3 *b*_3_). Subsequently, the external portion of the air bubble merge with the cavitation bubble and collapses (Figs. S3 *b*_4_-*b*_5_), while the portion inside the pit has not yet reached its minimum volume. At *t** = 1.159, the air bubble part trapped inside the pit contracts to its minimum volume (Fig. S3 *b*_7_), with a time interval of approximately 28 μs compared to the cavitation bubble contracts to its minimum volume. In Fig. S3(*c*) (*R*_max_ = 11.1 mm, *φ* = 0.55), the air bubble still splits into two parts during the cavitation bubble contraction (Fig. S3 *c*_3_). However, the external air bubble does not merge with the cavitation bubble, it collapses at *t** = 1.125 (Fig. S3 *c*_4_), the cavitation bubble itself collapses at *t** = 1.134 (Fig. S3 *c*_5_). Similarly, the internal portion of the air bubble collapses to its minimum volume later, at *t** = 1.150 (Fig. S3 *c*_7_). As the pit diameter (*φ*)increases further (Figs. S3 *d-f*), the air bubble no longer splits under the influence of the cavitation bubble. Furthermore, a larger pit diameter (*φ*)results in a smaller maximum expansion volume of the air bubble (as shown in Figs. S3 *d*_2_-*f*_2_).

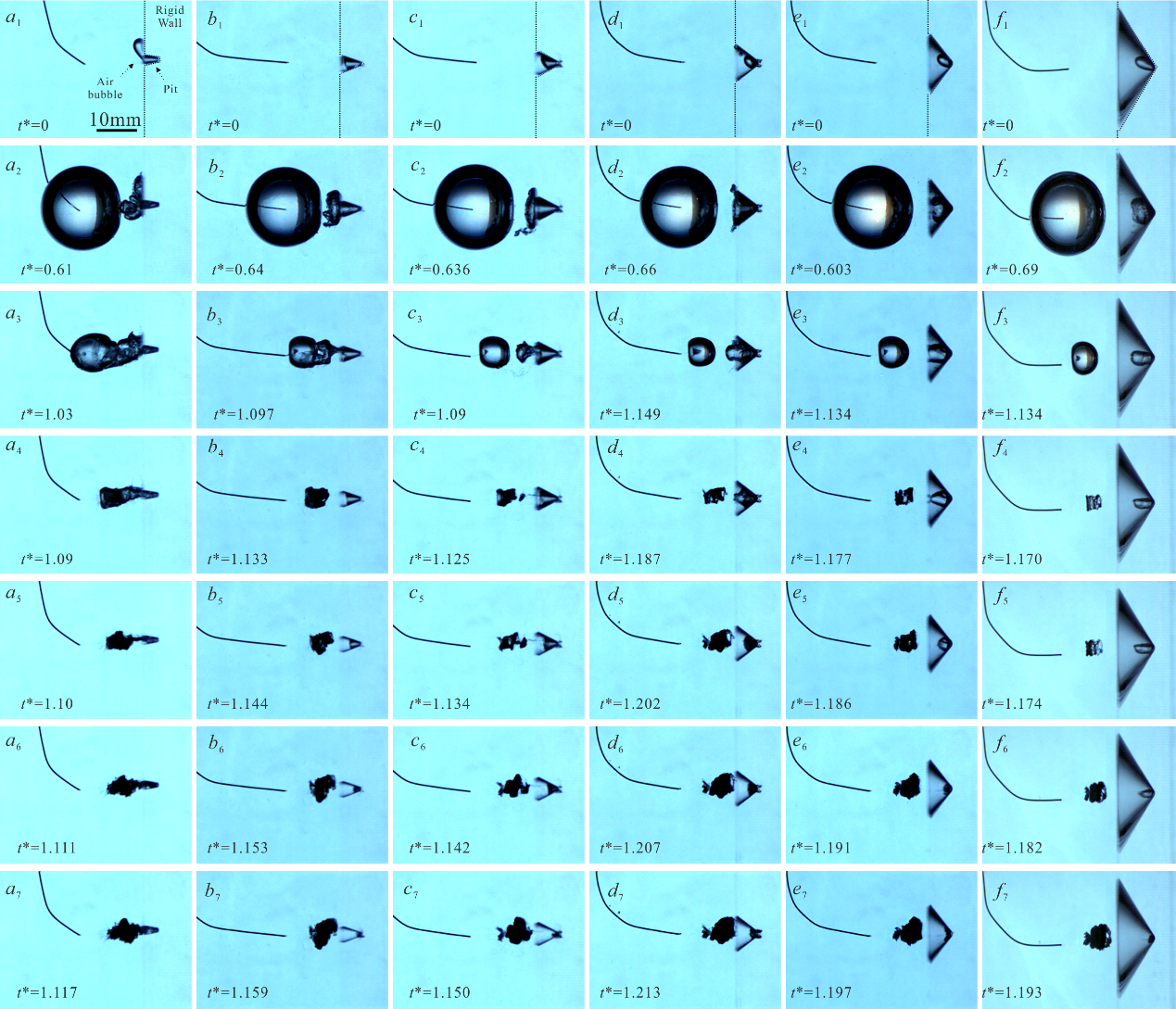


Fig.S3 Influence of pit diameter on the morphology of air bubble in pit (Exposure time:3.95 μs, frame rate: 180,000 fps; *r*_air_≈1.5mm, *η*=0.55, *γ*≈1.3; *a*: *R*_max_=11.05,*φ*=0.18; *b*: *R*_max_ =10.9, *φ*=0.37; *c*: *R*_max_ =11.1, *φ*=0.55; *d*: *R*_max_ =10.74, *φ*=0.83; *e*: *R*_max_ =10.69, *φ*=1.38; *f*: *R*_max_ =10.96, *φ*=2.95)

1. Contact author: luojing@scu.edu.cn [↑](#footnote-ref-1)
